# An inguinal hernia sac tumor of extrahepatic cholangiocarcinoma origin

**DOI:** 10.1186/1477-7819-4-13

**Published:** 2006-03-06

**Authors:** Naoyuki Yokoyama, Yoshio Shirai, Hidehiro Yamazaki, Katsuyoshi Hatakeyama

**Affiliations:** 1 Division of Digestive and General Surgery, Niigata University Graduate School of Medical and Dental Sciences, Asahimachi-dori 1-757, 951-8510, Niigata, Japan; 2 Department of Surgery, Niigata Minami Hospital, Meike-Shinmei 1-7-1, 950-8601, Niigata, Japan

## Abstract

**Background:**

Metastatic hernia sac tumor from biliary malignancy is extremely rare with only one such case previously reported. We herein report an additional case of extrahepatic cholangiocarcinoma presenting as a hernia sac tumor.

**Case presentation:**

A 78-year-old man presented with an irreducible right inguinal hernia associated with a firm tumor, 2.0 cm in diameter. A computed tomography scan demonstrated a soft tissue density mass with heterogeneous enhancement within the right inguinal canal. The patient underwent a hernia repair and the hernia sac tumor was resected. Histological examination of the tumor revealed a metastatic adenocarcinoma suggesting the tumor was of pancreato-biliary origin. Further investigation using imaging studies disclosed a primary tumor in the upper bile duct. The patient died of the disease nine months after the resection.

**Conclusion:**

Hernia sac tumors should be considered when an irreducible, growing mass appears within an inguinal hernia. Computed tomography may be useful for the early detection of hernia sac tumors from undiagnosed intra-abdominal malignancies.

## Background

Tumors associated with inguinal hernias are rare, occurring in less than 0.5% of surgically excised sacs [1]. Thus far, computed tomography (CT) features of hernia sac tumors have been poorly documented [2,3]. We herein report the case of a hernia sac tumor that originated from an extrahepatic cholangiocarcinoma, and specifically focus on the CT features exhibited by a metastatic tumor within an inguinal hernia.

## Case presentation

**Figure 1:**
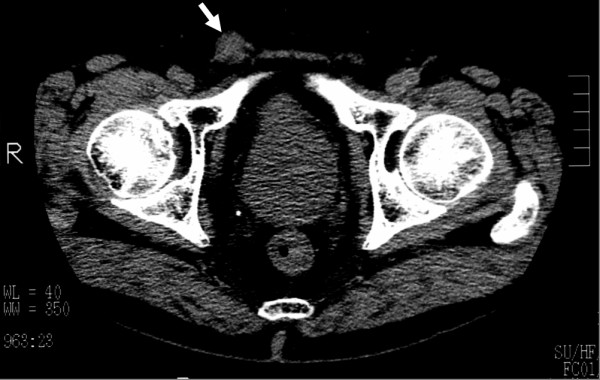
Plain computed tomography image showing a soft tissue density mass (arrow) within the right inguinal canal. The mass had no connection with intra-abdominal organs.

**Figure 2:**
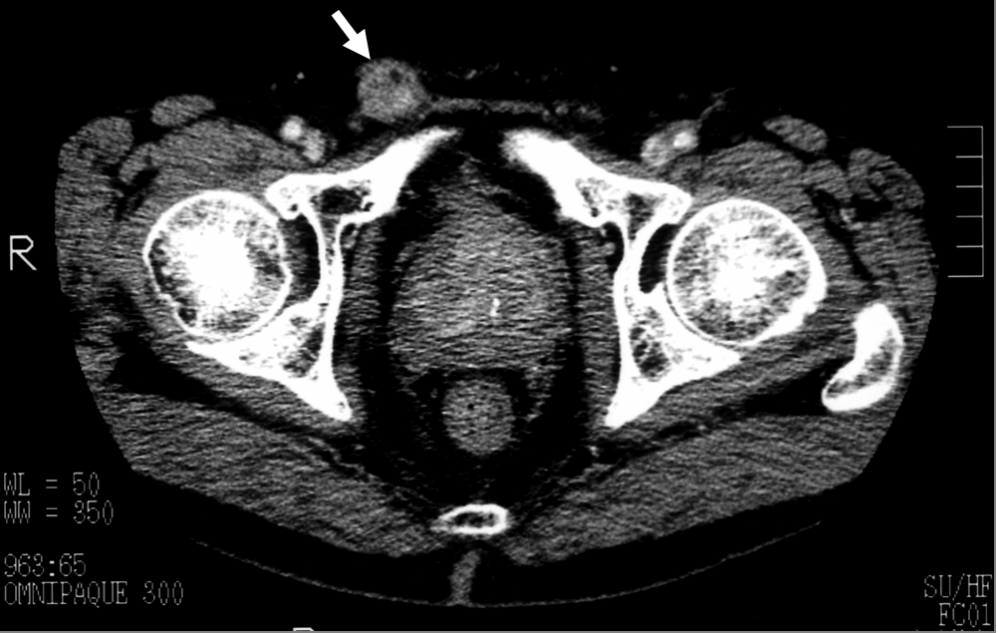
Contrast computed tomography scan image showing the inguinal tumor with heterogeneous enhancement (arrow).

A 78-year-old man presented with a tender mass in the right groin that had appeared five months earlier and had gradually grown. He had a three-year history of right inguinal hernia. Physical examination revealed an irreducible right inguinal hernia associated with a firm tumor, 2.0 cm in diameter. A plain CT scan demonstrated a soft tissue density mass within the right inguinal canal, which had no connection with intra-abdominal organs (Figure 1) and showed heterogeneous enhancement on a contrast CT scan (Figure 2). After being diagnosed with a hernia sac tumor of unknown origin, the patient underwent a hernia repair (iliopubic tract repair) under spinal anesthesia. A white tumor located at the tip of the sac encompassed the testicular artery and vein, and was resected together with the sac and the testicular vessels. Histological examination of the tumor revealed a metastatic, moderately differentiated adenocarcinoma with perineural invasion, suggesting that the tumor was of pancreato-biliary origin. Further investigation using abdominal CT scans and ultrasonography disclosed a tumor in the upper bile duct associated with gallbladder swelling. The primary tumor was diagnosed as an extrahepatic cholangiocarcinoma. Further investigations detected no malignancies in other organs. The patient died of the disease nine months after the operation despite systemic chemotherapy using gemcitabine. At the time of his death, neither hernia recurrence nor local tumor relapse were found in the right inguinal region.

## Discussion

Hernia sac tumors can be classified into one of three groups: *intrasaccular*, in which an organ bearing the tumor is incarcerated within the sac; *saccular*, in which the tumor encompasses the sac or spermatic cord structures; or *extrasaccular*, in which the tumor protrudes through the hernia defect but is located outside the sac [1]. Accordingly, the metastatic tumor described in this case can be classified as a saccular hernia sac tumor. The most common cause of saccular tumors is metastases from abdominal malignancies that migrate under the influence of gravity [1]. Hernia sac tumors are associated with a wide spectrum of tumor types. Among them, the most frequent primary site is the colon followed by the ovaries, prostate, pancreas, and appendix [1,4]. The hernia sac tumor described in this case originated from an extrahepatic cholangiocarcinoma, which is an extremely rare event with only one such case previously reported [4].

CT features of metastatic inguinal saccular tumors have been poorly documented, and to our knowledge there are only two documented cases that present CT findings of saccular tumors [2,3]. Yokota and colleagues reported that a saccular tumor of colonic carcinoma origin was depicted in a contrast CT scan as a soft tissue density mass with heterogeneous enhancement [2]. These CT scan findings are consistent with those described in the present case, suggesting that a soft tissue density mass with heterogeneous enhancement is a common CT feature of saccular tumors of gastrointestinal carcinoma origin. However, the contrast CT scan results for a saccular tumor originating from pseudomyxoma peritonei show a low-density mass without enhancement, suggesting a gelatinous composition [3]. CT scan findings from the present and previous studies highlight the potential use of preoperative CT scans for the differential diagnosis of hernia sac tumors.

## Conclusion

Hernia sac tumors can be suspected when an irreducible, growing mass appears within an inguinal hernia. Since hernia repair offers an opportunity for peritoneal biopsy, selective microscopic examination of grossly abnormal hernia sac specimens is recommended. Preoperative CT imaging may also be useful for the early detection of hernia sac tumors from undiagnosed intra-abdominal malignancies.

## Competing interests

The author(s) declare that they have no competing interests.

## Authors' contributions

**NY: **participated in the operation, searched the literature, and wrote the original manuscript.

**YS: **assisted in literature search and preparing the manuscript.

**HY: **participated in the operation and performed chemotherapy to the patients.

**KH: **supervised preparation of the manuscript and edited the final version for publication.

All authors read and approved the final manuscript.
